# Prognostic value of FDG-PET radiomics with machine learning in pancreatic cancer

**DOI:** 10.1038/s41598-020-73237-3

**Published:** 2020-10-12

**Authors:** Yoshitaka Toyama, Masatoshi Hotta, Fuyuhiko Motoi, Kentaro Takanami, Ryogo Minamimoto, Kei Takase

**Affiliations:** 1 Department of Diagnostic Radiology, Tohoku University Hospital, 1-1 Seiryo-machi, Aoba-ku, Sendai, 980-8574 Japan; 2 Division of Nuclear Medicine, Department of Radiology, National Center for Global Health and Medicine, 1-21-1 Toyama, Shinjuku-ku, Tokyo, 162-8655 Japan; 3 Department of Surgery 1, Yamagata University, 2-2-2 Iida-Nishi, Yamagata, 990-9585 Japan

**Keywords:** Cancer, Molecular medicine, Mathematics and computing

## Abstract

Patients with pancreatic cancer have a poor prognosis, therefore identifying particular tumor characteristics associated with prognosis is important. This study aims to investigate the utility of radiomics with machine learning using ^18^F-fluorodeoxyglucose (FDG)-PET in patients with pancreatic cancer. We enrolled 161 patients with pancreatic cancer underwent pretreatment FDG-PET/CT. The area of the primary tumor was semi-automatically contoured with a threshold of 40% of the maximum standardized uptake value, and 42 PET features were extracted. To identify relevant PET parameters for predicting 1-year survival, Gini index was measured using random forest (RF) classifier. Twenty-three patients were censored within 1 year of follow-up, and the remaining 138 patients were used for the analysis. Among the PET parameters, 10 features showed statistical significance for predicting overall survival. Multivariate analysis using Cox HR regression revealed gray-level zone length matrix (GLZLM) gray-level non-uniformity (GLNU) as the only PET parameter showing statistical significance. In RF model, GLZLM GLNU was the most relevant factor for predicting 1-year survival, followed by total lesion glycolysis (TLG). The combination of GLZLM GLNU and TLG stratified patients into three groups according to risk of poor prognosis. Radiomics with machine learning using FDG-PET in patients with pancreatic cancer provided useful prognostic information.

## Introduction

Pancreatic cancer is associated with poor prognosis^1^ and is the fourth most common cause of cancer death in Japan, the USA, and Europe^2–4^. Despite advances in the past decades in surgery, radiation therapy, and chemotherapy, the 5-year survival rate remains less than 9%^5^. Therefore, identifying particular tumor characteristics associated with poor prognosis is important at the initial assessment. Numerous 18F-fluorodeoxyglucose (FDG)-PET reports have demonstrated the efficacy of conventional PET features such as maximum standardized uptake value (SUVmax), metabolic tumor volume (MTV), and total lesion glycolysis (TLG) for predicting therapeutic response and prognosis^6–9^. However, those conventional PET features do not represent the spatial tumoral heterogeneity, which is deeply associated with cellular and molecular characteristics such as cellular proliferation and necrosis^10,11^.

Texture analysis has recently been identified as a volume-based method for quantifying tumor properties that are beyond the capability of visual interpretation or simple metrics as an essential tool for “radiomics”^12,13^. Radiomics is defined as the conversion of digital medical images into high-dimensional quantitative features, enabling data to be extracted and applied to the improvement of diagnostic and prognostic accuracy. This field has increased in importance for cancer research in recent years. Radiomics offers new opportunities for developing a better understanding of oncological processes, enabling personalized therapy^6,11^. Some recent radiomics studies have used machine-learning methods such as support vector machines, neural networks, and random forest (RF) classifiers^14–16^ that can improve the robustness of the statistical analysis^12^. However, few studies have explored the prognostic value of radiomics in pancreatic cancer using FDG-PET/CT with texture analysis^17–20^. To the best of our knowledge, no study has evaluated the prognostic value of FDG-PET/CT radiomics with machine learning in pancreatic cancer.

We hypothesized that radiomics with machine learning can provide a useful combination of clinical information, volume-based PET imaging parameters, and PET texture features that provide prognostic information for patients with pancreatic cancer. The aim of this study was to evaluate the prognostic value of FDG-PET radiomics with machine learning in pancreatic cancer.

## Results

A total of 161 patients were included in the analysis. Table 1 lists the patient demographics. The median follow-up period was 13.2 months (interquartile range 7.7–22.7 months), and median survival time was 16.9 months (95% CI 13.7–21.8 months). Twenty-three patients were censored within 1 year, and 138 patients (alive, n = 87; dead, n = 51) were used in the RF analysis.

**Table 1:** Patient demographics.

Patients number	161
**Sex (%)**	
Male	94 (58.4)
Female	67 (41.6)
Mean age (years) (SD)	65.98 (10.13)
Mean BMI (kg/m^2^) (SD)	22.42 (3.51)
**Tumor location (%)**	
Head	96 (59.6)
Body	41 (25.5)
Tail	24 (14.9)
Median tumor size [IQR]	32.00 [25.00, 41.00]
**Clinical stage (%)**	
I	73 (45.3)
II	36 (22.4)
III	24 (14.9)
IV	28 (17.4)
T stage (%)*	
1	24 (14.9)
2	75 (46.6)
3	27 (16.8)
4	35 (21.7)
**Regional lymph node metastasis (%)**	
Negative	112 (69.6)
Positive	49 (30.4)
**Distant metastasis (%)**	
Negative	133 (82.6)
Positive	28 (17.4)
Median CA19-9 [IQR]	166.90 [43.90, 904.70]
Median CEA [IQR]	3.70 [2.00, 6.30]
**Treatment (%)**	
Surgical treatment	75 (46.6)
Non-surgical treatment (chemo and/or radiation)	84 (53.4)

*SD* standardized deviation, *BMI* body mass index, *IQR* interquartile range.

*According to the 8th edition of the TNM classification of malignant tumors.

### Univariate and multivariate Cox hazard regression analysis

**Figure 1:**
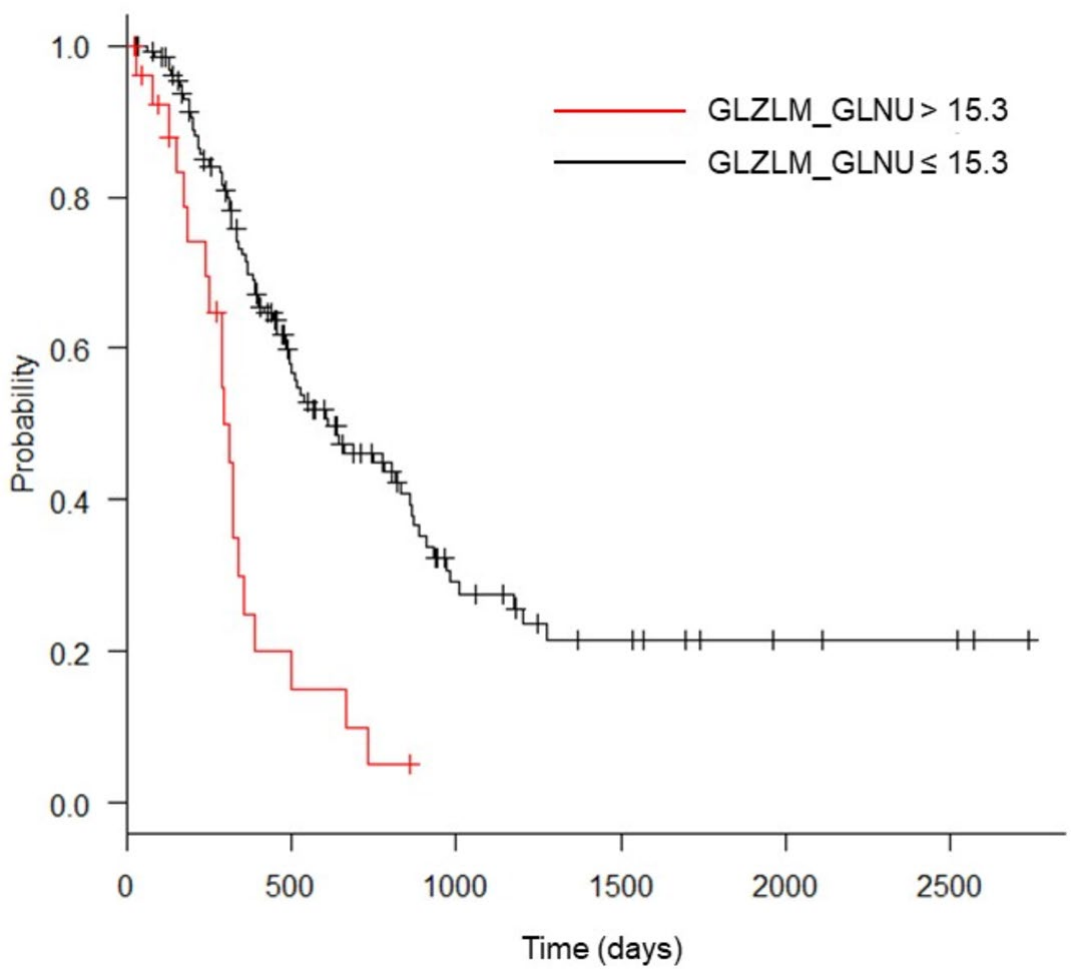
Kaplan–Meier curves of overall survival of patients for gray-level zone length matrix (GLZLM) zone-length non-uniformity (GLNU) > 15.3 and GLZLM GLNU ≤ 15.3.

Among the clinical characteristics, clinical stage and surgical treatment were identified as significantly important factors for predicting overall survival (Table 2). Among the PET parameters, 10 features showed statistical significance (log-rank p < 0.001) for predicting overall survival; of these, multivariate analysis with Cox HR regression revealed gray-level zone length matrix (GLZLM) gray-level non-uniformity (GLNU) as the only statistically significant PET parameter (Table 3). Kaplan–Meier curves for GLZLM GLNU are shown in Fig. 1.

**Table 2:** Univariate Cox hazard regression analysis of the clinical characteristics.

	Hazard ratio (95% CI)	p value
Sex (male)	1.0 (0.7–1.5)	0.95
Age (> 60 years)	1.5 (0.9–2.3)	0.081
BMI (> 22 kg/m^2^)	1.0 (0.7–1.5)	0.95
Clinical stage (III, IV)	2.0 (1.3–3.0)	0.0018
CA19-9 (> 213.6)	1.5 (0.9–2.6)	0.16
CEA (> 2.8)	0.9 (0.5–1.6)	0.76
Treatment (surgical treatment)	0.23 (0.15–0.37)	< 0.001

**Table 3:** Univariate and multivariate Cox hazard ratio regression analyses of PET features that showed statistical significance (p < 0.0018) in Kaplan–Meier analysis with the log-rank test.

PET features (optimal cutoff value)	Univariate		Multivariate	
	Hazard ratio (95% CI)	p value	Hazard ratio (95% CI)	p value
MTV (> 10.7)	2.9 (1.6–5.4)	< 0.001	0.8 (0.3–2.3)	0.66
TLG (> 34.6)	4.4 (2.0–9.6)	< 0.001	2.1 (0.7–6.6)	0.21
SHAPE_Sphercity (< 96.8 × 10^–2^)	2.2 (1.4–3.2)	< 0.001	1.2 (0.7–2.0)	0.52
SHAPE_Compacity (< 16.1 × 10^–2^)	2.3 (1.5–3.7)	< 0.001	1.0 (0.5–1.9)	0.97
NGLDM_Coarseness (< 16.3 × 10^–3^)	2.6 (1.7–4.1)	< 0.001	1.43 (0.7–2.8)	0.29
GLRLM_LGRE (< 16.2 × 10^–3^)	5.4 (1.3–22.0)	0.018		
GLRLM_RLNU (> 21.2 × 10)	4.4 (2.0–9.5)	< 0.001	2.1 (0.5–8.4)	0.31
GLRLM_SRLGE (< 10.0 × 10^–3^)	2.1 (1.3–3.6)	0.003		
GLZLM_GLNU (> 15.3)	3.2 (1.9–5.3)	< 0.001	2.1 (1.2–3.9)	0.011
GLZLM_LGZE (< 12.5 × 10^–3^)	2.2 (1.2–3.8)	0.008

### Machine learning analysis

**Figure 2:**
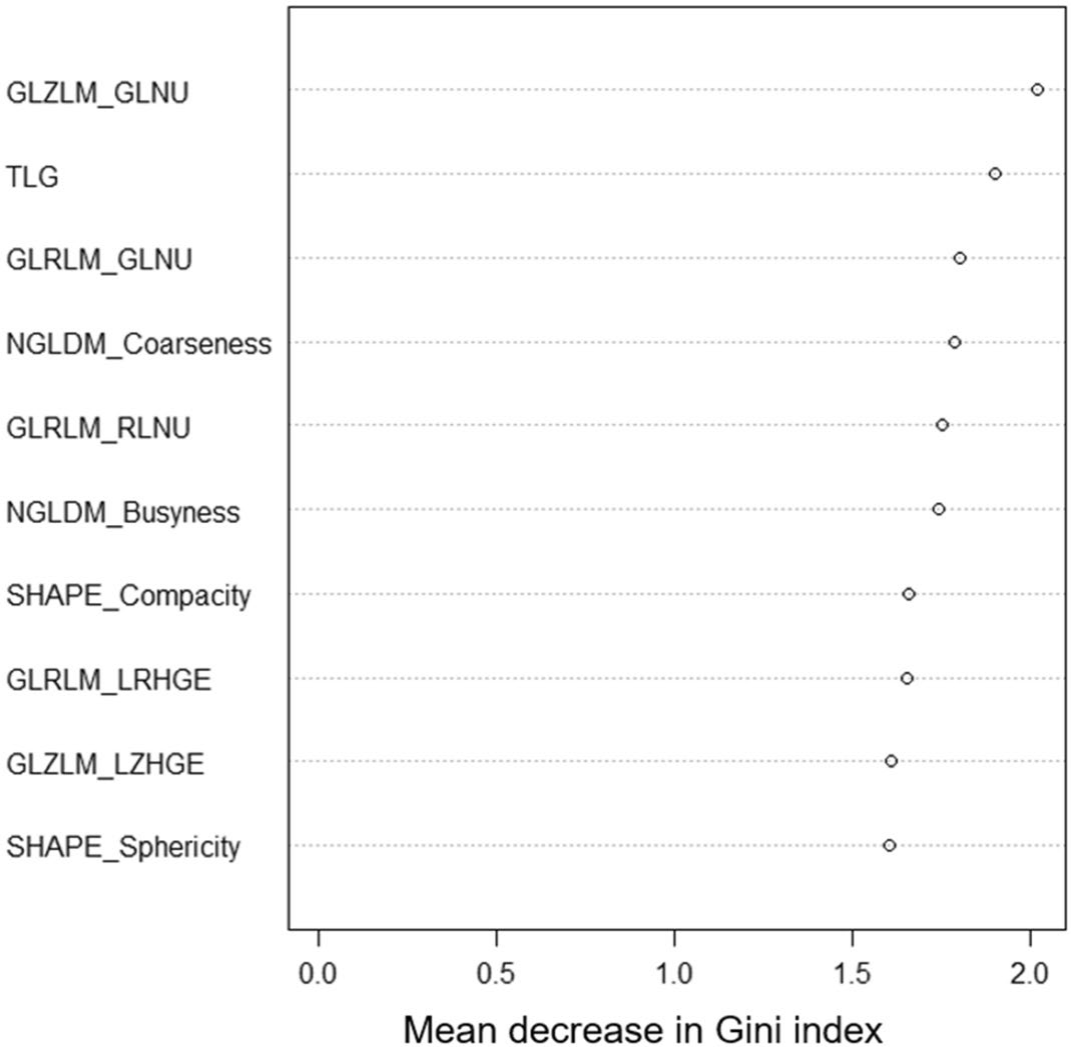
The top 10 PET parameters for predicting survival according to mean decrease in Gini index evaluated by random forest classifier. *GLZLM* gray-level zone length matrix, *GLNU* zone-length non-uniformity, *TLG* total lesion glycolysis, *GLRLM* gray-level run length matrix, *NGLDM* neighborhood gray-level different matrix, *RLNU* run length non-uniformity, *LRHGE* long-run high gray-level emphasis, *LZHGE* large-zone high gray-level emphasis.

**Figure 3:**
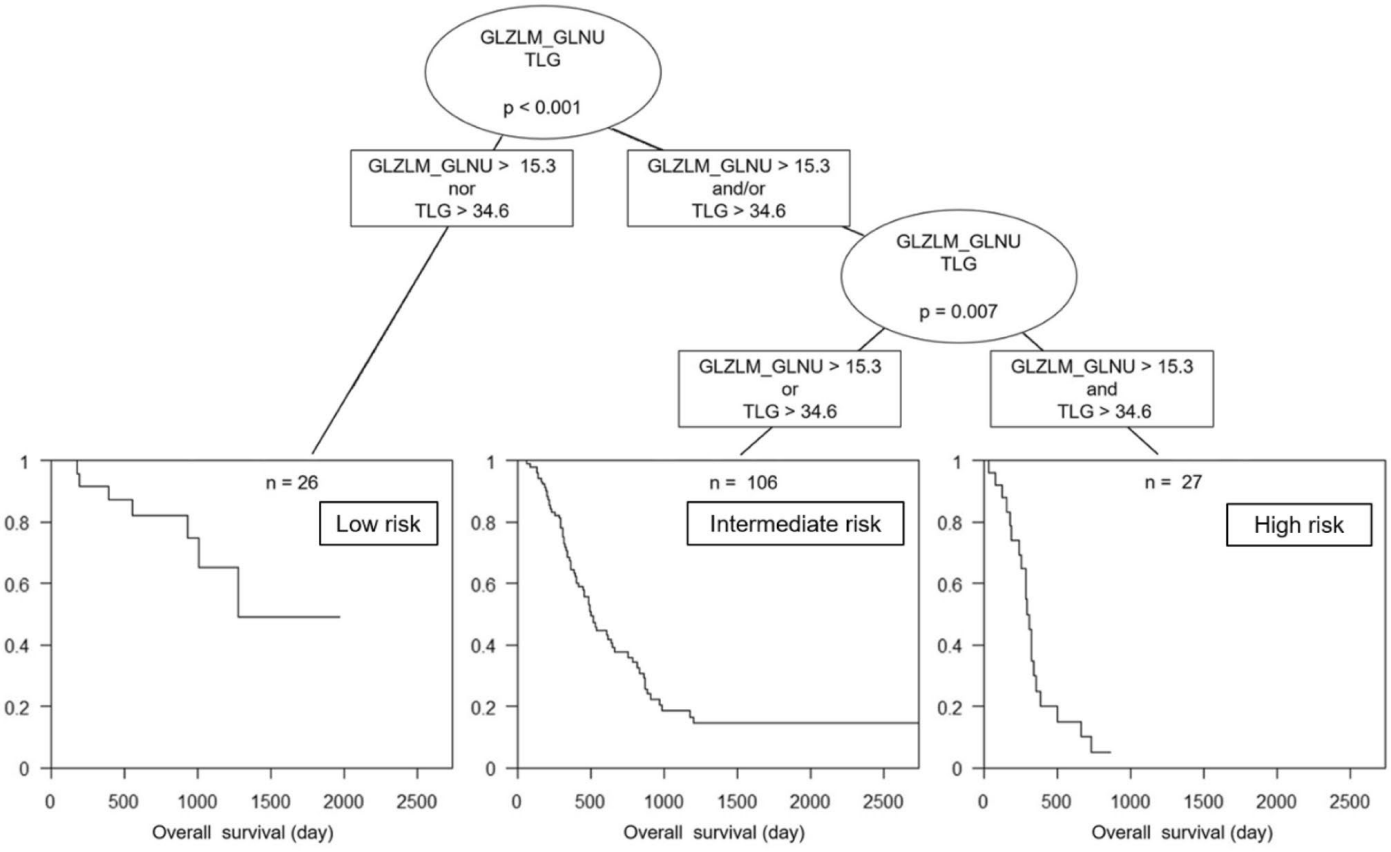
Decision-tree-based classification of patients for poor prognosis using gray-level zone length matrix (GLZLM) zone-length non-uniformity (GLNU) and total lesion glycolysis (TLG).

GLZLM GLNU was an independent risk factor for poor prognosis regardless of clinical stage and surgical status (Table 4). In the RF model, GLZLM GLNU was the most relevant factor for predicting 1-year survival, followed by total lesion glycolysis (TLG) (Fig. 2). The combination of GLZLM GLNU and TLG appropriately stratified patients into three groups according to risk for poor prognosis (Fig. 3). This combination was also effective in a subgroup analysis of patients who had received surgical treatment alone (Supplemental Figure S1).

**Table 4:** Multivariate Cox hazard ratio regression analysis of gray-level zone length matrix (GLZLM) gray-level non-uniformity (GLNU), surgical treatment, and clinical stage.

	Multivariate	
	Hazard ratio (95% CI)	p value
GLZLM_GLNU (> 15.3)	2.0 (1.2–3.4)	0.0094
Treatment (surgical treatment)	0.29 (0.18–0.48)	< 0.001
Clinical stage (III, IV)	1.3 (0.8–2.0)	0.23

## Discussion

The present study appears to be the first to evaluate the prognostic value of FDG-PET radiomics with machine learning in pancreatic cancer. Among the various PET parameters, GLZLM GLNU was the most relevant feature for predicting prognosis in multivariate analysis and machine learning analysis with RF. In addition, GLZLM GLNU combined with TLG, which was the second most important factor in the RF model, enabled stratification of patients into three groups according to their risk for poor prognosis.

We selected an RF classifier for use in a machine-learning approach. Random forest is an ensemble approach that computes multiple decision-tree-based classifiers using implicit feature selection^21^. Although a number of studies of malignant diseases have reported the clinical implications of intratumoral heterogeneity on FDG-PET, a lack of standardization complicates the comparison of these results. In their critical review, Hatt et al. described common issues in recent studies of texture analysis such as variability of nomenclature, workflow complexity, and redundancy of features; moreover, they recommended using robust machine-learning techniques to achieve better redundancy analysis and feature selection/combination^12^. Among the various machine-learning techniques, the advantage of RF in being able to predict features non-parametrically even if some features show collinearities with others suggests its suitability for texture analysis. Indeed, Ahn et al. reported that an RF classifier provided higher diagnostic performance compared with other machine-learning algorithms, including support vector machine and neural network algorithms, for predicting the prognosis of lung cancer on FDG-PET^14^. The RF classifier technique shows promise for extraction of the most prognostic PET features.

In multivariate analysis, GLZLM GLNU was the only PET parameter that showed statistical significance, and was the most important factor for predicting prognosis in the RF model, outperforming conventional FDG-PET parameters such as SUVmax and metabolic tumor volume. The gray-level zone length matrix (GLZLM, also termed gray level size zone matrix [GLSZM]) is a regional textural feature. It provides information regarding the size of homogeneous zones for each gray level in three dimensions. Gray-level non-uniformity (GLNU) is a measure of the similarity of gray-level values throughout the image^22^; as with many other textural features, the value of GLSZM GLNU increases if the lesion is heterogeneous^23–25^. Intratumoral heterogeneity is associated with tumor aggressiveness, treatment response, and prognosis^12,26^. Many studies have demonstrated the clinical value of PET radiomics with textural features for various malignancies^27^; however, few have investigated the clinical value of PET radiomics with textural features in pancreatic cancer^17–20^. These studies were all were FDG-PET-based, and primarily assessed the prognostic value of intratumoral heterogeneity for predicting survival. Hyun et al. investigated the utility of texture analysis on FDG-PET in 137 patients with pancreatic cancer who underwent diverse treatment and supportive care. In time-dependent ROC curve analysis for 2-year survival prediction, entropy (a global textural feature) and heterogeneity index showed the highest AUC value (0.720), followed by TLG (AUC = 0.697)^18^. In the present study, “entropy” corresponds to “Entropy(log2)” in the Global-textural Histogram and was ranked 23rd out of 42 features in the RF analysis (Supplemental Table S1), but direct comparisons are difficult to make because the present study deals with a larger number of features than did previous studies (36 features). Furthermore, the present study included many patients with stage 1 pancreatic cancer (45%). Although possibly the cause of the difference in results compared with the study of Hyun et al. (no stage 1), it provides an advantage in predicting patient prognosis at an early stage. Although there are subtle differences in the feature types, the results are consistent with our findings, in that textural features reflecting intratumoral heterogeneity and the volumetric parameter TLG are the two most important prognostic factors. As well as being complementary, intratumoral heterogeneity by texture analysis and conventional volumetric PET parameters in combination enable more accurate prognostic analysis in pancreatic cancer.

The results of the present study revealed surgical treatment as the strongest prognostic factor among the clinical features; however, we do not have this important information at the point of clinical decision making. In addition, GLZLM GLNU was identified as an independent risk factor for poor prognosis regardless of surgical treatment, and high GLZLM GLNU and/or TLG were associated with worse survival in patients who had undergone surgery, and also in the overall patients. The use of these imaging biomarkers could help improve risk stratification and enhance cancer management.

Several limitations must be considered in this study. First, this was a retrospective study in which the patients had undergone various treatment protocols. All patients underwent FDG-PET prior to any treatment, but had different clinical courses. Second, this was a single-center study; nevertheless, it included a relatively large number of patients compared with previous studies. Our study results need to be validated in a prospective multi-center study with external data. Third, lesions without significant uptake were excluded from analysis. This limitation is not specific to our study, and is inevitable in appropriate texture analysis^28^.

In conclusion, radiomics with machine learning using FDG-PET in pancreatic cancer extracted factors of useful prognostic value; in particular, the combination of GLZLM GLNU and TLG appropriately stratified patients according to their risk for poor prognosis. This information could be beneficial in pretreatment clinical decision making in patients with pancreatic cancer, enabling personalized medicine such as risk-based follow-up and enhanced chemotherapy. Further prospective validation studies are required before FDG-PET radiomics with machine learning can be applied to practical clinical use.

## Methods

### Patients

**Figure 4:**
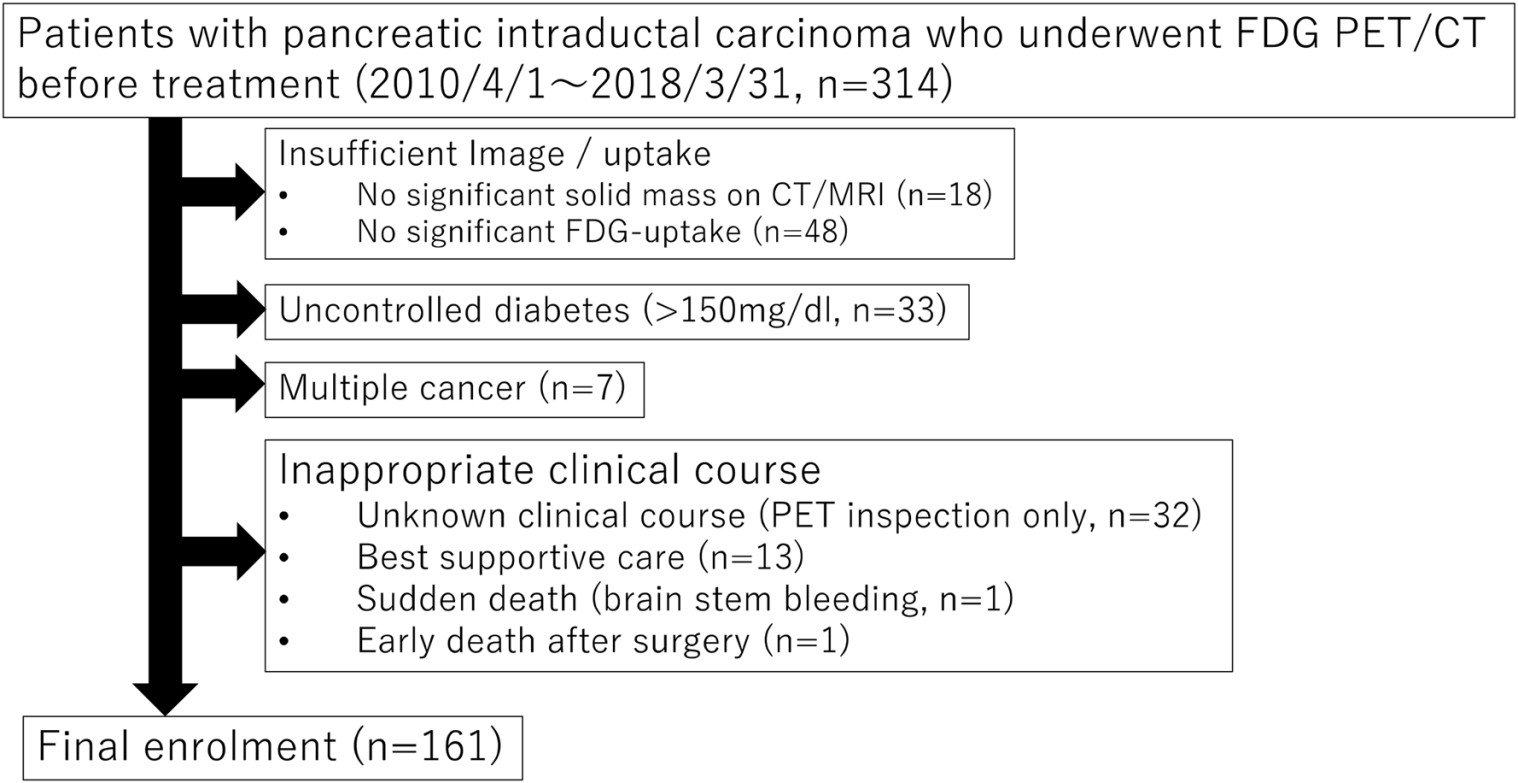
Flowchart of patient selection.

This retrospective study was approved by our institutional Ethics Review Board (Independent Ethics Committee of Tohoku University School of Medicine) and the requirement to obtain informed consent from participants was waived due to the retrospective nature of the investigation. We enrolled 314 consecutive patients with biopsy-confirmed pancreatic invasive ductal carcinoma who underwent FDG-PET/CT before treatment between April 2010 and March 2018. The exclusion criteria were as follows: (1) no significant solid mass on CT/MRI (n = 18); (2) no significant FDG-uptake (n = 48); (3) uncontrolled diabetes (< 150 mg/dl; n = 33); (4) multiple cancer (n = 7); (5) unknown clinical course (n = 32); (6) under best supportive care (n = 13); (7) sudden death (brain stem bleeding; n = 1); (8) early death after surgery (n = 1) (Fig. 4). All patients received surgery, chemotherapy, radiation therapy, or combination therapy of these.

### PET/CT acquisition

After a 4-h fast, all patients were injected with FDG (3.7 MBq FDG/kg body weight) 60 min before initiating the PET/CT scan (Biograph 40, Siemens Medical Solutions, Erlangen, Germany). Spiral CT data were acquired from the thigh to the top of the skull with ~ 25 effective mAs, 130 kVp, and 5-mm slice thickness, and CT images were used for attenuation correction as well as image fusion. PET images of the same area were acquired in three-dimensional mode with 2 min per bed position, and reconstructed with an ordered subset expectation maximization algorithm (6 iterations and 14 subsets) to a final pixel size of 4.1 mm. An 8-mm full-width at half maximum Gaussian filter was used as a post-smoothing filter.

### Radiomic feature extraction

To obtain the volume of interest (VOI) of the primary tumor, a sphere was set to encompass the lesion and then contoured using a threshold of 40% of the SUVmax (Supplemental Figure S2). A total of 42 PET parameters (Supplemental Table S1) including conventional features (e.g., SUVmax, MTV, TLG) and global, local, and regional texture features were measured using the LIFEx package^29^. Texture features were calculated only for VOIs of ≥ 64 voxels because textural features cannot be accurately quantified for small regions^28^. All PET/CT images were assessed by two nuclear medicine physicians (M.H. and Y.T, with 12 and 10 years of experience in CT and 5 and 4 years of expertise in PET, respectively), with decisions made in consensus. In cases of disagreement, a final consensus was achieved by discussion.

The study endpoint was overall survival (OS), defined as the time from pretreatment FDG-PET/CT scan to cancer-related death. Outcome data were collected from the medical records of each patient. Surviving patients were censored at the time of last clinical follow-up.

### Machine learning and statistical analysis

All statistical analyses were performed using R version 3.5.1 (R Foundation for Statistical Computing, Vienna, Austria). Kaplan–Meier analysis with the log-rank test was performed for PET parameters and clinical features. Optimal cutoff values of the PET parameters were obtained by Classification and Regression Tree (CART) analysis using the “rpart” R package. CART is a tree-building-based technique in which several predictor variables are tested to determine their impact on such as including overall survival^30^. The cutoff values for age and BMI were set at 60 years and 22 kg/m^2^, respectively, based on their clinical importance. Receiver-operating characteristic (ROC) analysis was performed to identify the optimal cutoff values for tumor markers CA19-9 and CEA. For PET parameters, the p value threshold for statistical significance was set at < 0.0012 (0.05/42) following Bonferroni correction. For the other analyses, p values < 0.05 were regarded as significant. Univariate and multivariate analyses were performed using Cox hazard ratio (HR) regression. To identity the PET parameters important for prediction of 1-year survival, mean decrease in Gini index was evaluated using an RF classifier with “randomForest” R package, in the population excluding patients who had been censored less than 1 year. Random forest is an ensemble technique that computes multiple decision-tree-based classifiers using implicit feature selection. Gini index is an efficient approximation of entropy in a computational manner. It is calculated at each node split of the RF and reflects how well the data could be split into two classes at a particular node in each tree. Gini index measures the degree or probability of a particular variable being wrongly classified for each feature at a node^21,31^. The RF classifier was optimized for the number of trees (ntree) (100, 250, 500, 750, 1000, 1500) with repeated (n = 100) and tenfold cross-validation using the “caret” R package, and optimal ntree and number of variables tried at each split (mtry) were determined (ntree = 750, mtry = 1). Using the two most relevant PET parameters from the RF model, CART analysis was performed to classify patients into subgroups according to their risk for overall survival.

### Ethical statement

This study was approved by the local Ethics Committee and was carried out in accordance with the principles of the 1964 Declaration of Helsinki.

## Supplementary information

Supplementary Information 1
