# Supplementary material for: Prognostic value of FDG-PET radiomics with machine learning in pancreatic cancer
Source: Sci Rep. 2020 Oct 12;10:17024. doi: 10.1038/s41598-020-73237-3 (PMC7550575; doi:10.1038/s41598-020-73237-3)
Supplement: Supplementary file 1 — Supplementary Information 1 [file 41598_2020_73237_MOESM1_ESM.docx]

**Title**

Prognostic value of FDG-PET radiomics with machine learning in pancreatic cancer

**Authors**

Yoshitaka Toyama M.D.^1^, Masatoshi Hotta M.D.^2^, Fuyuhiko Motoi M.D.^3^, Ph.D., Kentaro Takanami M.D., Ph.D.^1^, Ryogo Minamimoto M.D., Ph.D.^2^, Kei Takase M.D.^1^

^1^ Department of Diagnostic Radiology, Tohoku University Hospital, 1-1 Seiryo-machi, Aoba-ku, Sendai, 980-8574, Japan.

^2^ Division of Nuclear Medicine, Department of Radiology, National Center for Global Health and Medicine, 1-21-1, Toyama, Shinjuku-ku, Tokyo, 162-8655, Japan.

^3^ Department of Surgery 1, Yamagata University, 2-2-2, Iida-Nishi, Yamagata, 990-9585, Japan.

**Corresponding author:**

Yoshitaka Toyama, Phone: +81-22-717-7312, Fax: +81-22-717-7316, E-mail: ytoyama0818@gmail.com

ORCID: 0000-0003-0027-9681

**Supplemental figure and table legends**

**
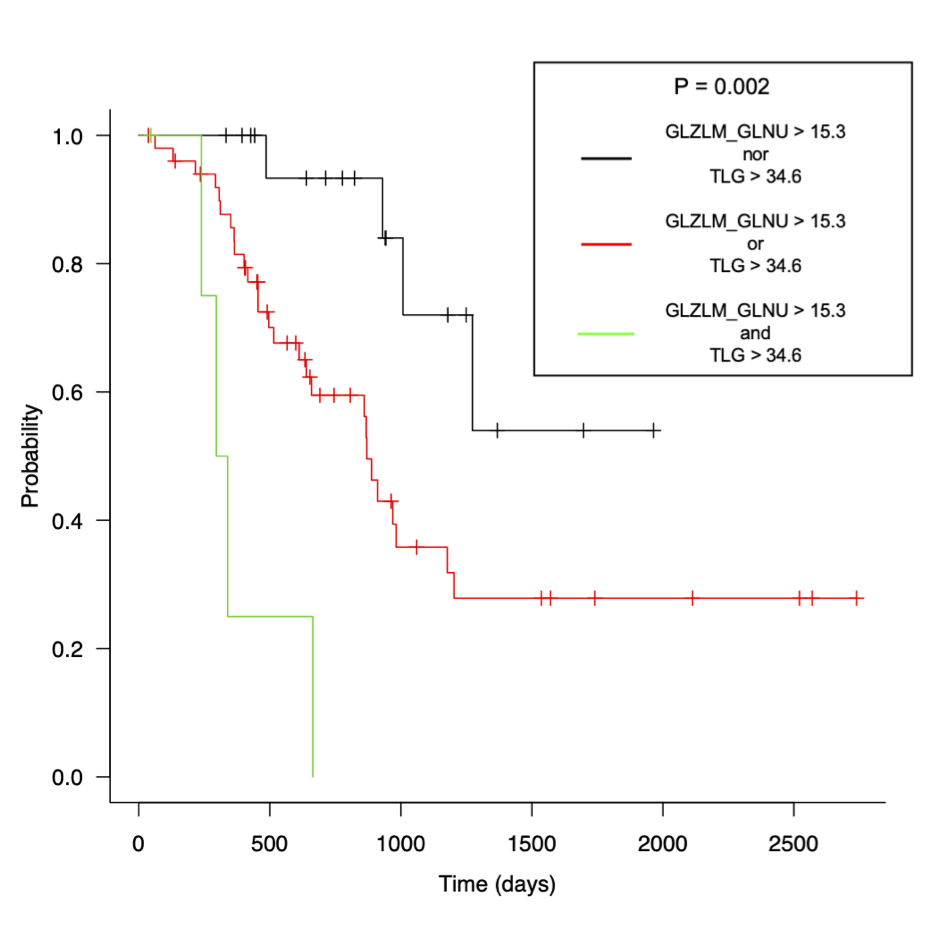
**

**Supplemental Figure 1.** Kaplan–Meier curves for overall survival of patients treated with surgery (n=75) divided into the following three groups: gray-level zone length matrix (GLZLM) zone-length non-uniformity (GLNU) > 15.3 nor total lesion glycolysis (TLG) > 34.6 (n=19), GLZLM GLNU > 15.3 or TLG >34.6 (n=51), GLZLM GLNU > 15.3 and TLG > 34.6 (n=5).

**Supplemental Figure 2.**

A 74-year-old man with pancreatic cancer. A) FDG-PET/CT shows an FDG-avid primary tumor. B) A sphere encompassing the lesion is set. C) A volume of interest is then obtained using a threshold that is 40% of the maximum standardized uptake value, which is used in texture analysis.

| Supplemental table 1. PET features used in random forest analysis | | |
| --- | --- | --- |
| Conventional |  | SUVmax, SUVmean, SUVstd, MTV, TLG |
| Shape |  | Sphericity, Compacity |
| Global-textural | Histogram | Energy, Entropy (log2), Kurtosis, Skewness |
| Local-textural | GLCM | Contrast, Correlation, Dissimilarity, Energy, Entropy (log2), Homogeneity |
|  | NGLDM | Busyness, Contrast, Coarseness |
| Regional-textural | GLRLM | Gray-Level Non-Uniformity, High Gray-Level Run Emphasis, Low Gray-Level Run Emphasis, Long-Run Emphasis, Long-Run High Gray-Level Emphasis, Long-Run Low Gray-Level Emphasis, Run Length Non-Uniformity, Run Percentage, Short-Run Emphasis, Short-Run High Gray-Level Emphasis, Short-Run Low Gray-Level Emphasis |
|  | GLZLM | Gray-Level Non-Uniformity, High Gray-Level Zone Emphasis, Low Gray-Level Zone Emphasis, Long-Zone Emphasis, Large-Zone High Gray-Level Emphasis, Large-Zone Low Gray-Level Emphasis, Small-Zone Emphasis, Short-Zone High Gray-Level Emphasis, Short-Zone Low Gray-Level Emphasis, Zone-Length Non-Uniformity, Zone Percentage |

**Supplemental Table 1.** PET features used in random forest analysis

*SUVmax* maximum standardized uptake value, *SUVmean* mean maximum standardized uptake value, *SUVstd* standard deviation of standardized uptake value, *MTV* metabolic tumor volume, *TLG* total legion glycolysis, *GLCM* gray-level co-occurrence matrix, *NGLDM* neighborhood gray-level different matrix, *GLRLM* gray-level run length matrix, *GLZLM* gray-level zone length matrix
